# Neutrophil extracellular trap-associated responses are associated with tissue factor expression and coagulation abnormalities during anaphylaxis

**DOI:** 10.3389/fimmu.2026.1905112

**Published:** 2026-08-25

**Authors:** Yuzhou Huang, Shenming Xu, Pengfei Rong, Dan Wang, Jianyun Lu

**Affiliations:** 1 Department of Dermatology, Third Xiangya Hospital, Central South University, Changsha, China; 2 Medical Ozone Research Center, Central South University, Changsha, China; 3 Department of Radiology, The Third Xiangya Hospital Central South University, Changsha, China

**Keywords:** anaphylaxis, neutrophil extracellular traps, NET-associated responses, tissue factor, coagulation abnormalities

## Abstract

**Background:**

Anaphylaxis is a severe, fast-onset hypersensitivity reaction. Studies have shown that neutrophils play an important role in the acute phase of anaphylaxis. Activated neutrophils can release neutrophil extracellular traps (NETs); however, the potential involvement of NET-associated responses in anaphylaxis and their relationship with coagulation abnormalities remain unclear.

**Objective:**

The aim of this study was to investigate the potential involvement of NET-associated responses in anaphylaxis and their association with tissue factor (TF) expression and coagulation abnormalities.

**Methods:**

NET-associated markers, inflammatory mediators, coagulation-related parameters, and indices of anaphylactic responses were assessed in patients with anaphylaxis, passive systemic anaphylaxis (PSA) and passive cutaneous anaphylaxis (PCA) mouse models, and IgE-stimulated primary neutrophils. DNase I was used to degrade extracellular DNA structures, and tissue factor pathway inhibitor (TFPI) was used to modulate TF-related coagulation signaling. Heparin sodium and dexamethasone (DXM) were administered to evaluate whether anticoagulant or anti-inflammatory treatment was accompanied by changes in NET-associated and coagulation-related markers.

**Results:**

Bioinformatics analysis identified NET-associated genes and related biological pathways in anaphylaxis. Increased NET-associated markers were detected in patients with anaphylaxis, PSA mice, and IgE-treated primary neutrophils. DNase I treatment reduced NET-associated markers, inflammatory cytokine levels, and the experimental anaphylactic responses. Mast-cell degranulation and NET-associated extracellular DNA signals were increased in PCA mice. Elevated TF levels were detected in patients with anaphylaxis, and increased TF expression was observed in neutrophils from PSA mice. TFPI treatment was associated with reduced TF expression, inflammatory cytokine expression, and NET-associated markers in experimental models. Among 323 patients, higher D-dimer and fibrin degradation product levels were associated with greater anaphylaxis severity, and D-dimer levels were positively correlated with neutrophil counts. DNase I and TFPI treatment reduced D-dimer and fibrin degradation product levels in PSA mice. Heparin sodium and DXM attenuated experimental anaphylactic responses and were accompanied by reductions in NET-associated, TF-related, and coagulation markers.

**Conclusions:**

NET-associated markers were increased in anaphylaxis and were associated with TF expression, coagulation abnormalities, and disease severity. Extracellular DNA degradation and TF pathway modulation were associated with attenuation of experimental anaphylactic responses. These findings support a potential association among NET-associated responses, TF expression and coagulation abnormalities, and anaphylaxis, although further mechanistic studies are required to establish direct causality.

## Introduction

Anaphylaxis is a rapidly progressing, life-threatening reaction typically triggered by medications, foods, or insect stings (1). It may present with respiratory distress, angioedema, cardiovascular compromise, and other rapidly evolving systemic manifestations. The incidence of anaphylaxis appears to be increasing worldwide, and its prevalence in the United States has been estimated at 1.6%–5.1%, with mortality rates among hospitalized patients ranging from 0.5% to 1% (2–5). Anaphylaxis can arise through immune-mediated or non-immune mechanisms, of which immunoglobulin E (IgE)-mediated activation of mast cells and basophils is the most common (6). IgE-dependent activation induces the rapid release of histamine, proteases, lipid mediators, and cytokines, resulting in widespread vascular, respiratory, and cardiovascular effects (7). Despite extensive research, the mechanisms underlying the progression from an acute allergic reaction to severe systemic anaphylaxis remain incompletely understood.

Neutrophils are essential and potent phagocytes of the innate immune system, involved in a range of diseases including infections, autoimmune diseases, cancer, and chronic inflammation (8). One exploratory study in an emergency setting suggested that the innate immune system, particularly neutrophils, may participate in the acute phase of anaphylaxis (9, 10). Under certain inflammatory conditions, activated neutrophils can release extracellular DNA structures commonly referred to as neutrophil extracellular traps (NETs), contain DNA decorated with histones and granular proteins. Excessive accumulation of NET-associated structures has been linked to tissue inflammation and disease progression (11, 12). NET-associated structures have been reported to interact with macrophages and other inflammatory cells, promote inflammatory mediator release, and contribute to amplification of inflammatory responses (13, 14). Increased NET-associated responses have also been reported in asthma, rheumatoid arthritis, septic lung injury, and ulcerative colitis, and have been investigated as markers of disease activity and clinical outcome (15, 16). Therefore, whether NET-associated responses contribute to the development of anaphylaxis remains to be clarified.

Tissue factor (TF) is the principal initiator of the extrinsic coagulation pathway. Exposure of blood to TF expressed on activated or damaged cells and TF-bearing extracellular vesicles initiate coagulation factor activation and promotes thrombin generation. Increasing evidence suggests that coagulation pathways are involved in allergic and inflammatory disorders. Alterations in TF, fibrinogen, factor XII, and related hemostatic pathways have been reported in asthma, chronic spontaneous urticaria, and mast cell-associated inflammatory responses (17, 18). NET-associated structures have also been linked to thrombo-inflammatory processes through interactions with platelets, coagulation factors, and TF expression (19). However, whether NET-associated responses are involved in TF expression and coagulation abnormalities during anaphylaxis remains unclear.

Therefore, this study investigated NET-associated responses in patients with anaphylaxis, mouse models of passive cutaneous and passive systemic anaphylaxis, and IgE-stimulated primary neutrophils. We further examined the associations among NET-associated markers, TF expression, coagulation-related parameters, and anaphylaxis severity, and examined whether modulation of extracellular DNA-associated processes and TF-related pathways was accompanied by changes in experimental anaphylaxis.

## Materials and methods

### Microarray dataset and bioinformatics analysis

The GSE69063 microarray dataset was downloaded from the Gene Expression Omnibus (GEO) database (https://www.ncbi.nlm.nih.gov/geo/) (20) using the GEOquery package (21). The dataset contains peripheral blood samples from patients with moderate (n = 6) and severe (n = 12) anaphylaxis and age- and sex-matched control cohorts (n = 10). Differential expression analysis was performed using the limma package. Differentially expressed genes (DEGs) were identified using the thresholds of |log_2_ fold change| > 1 and adjusted P < 0.05 (Benjamini–Hochberg false discovery rate, FDR).

NET-associated candidate genes were retrieved from the GeneCards (22) and MSigDB (23) databases using the keyword “neutrophil extracellular traps”. Candidate genes identified in both databases were intersected with DEGs to obtain NET-associated differentially expressed genes in anaphylaxis.

Gene Ontology (GO) (24), Kyoto Encyclopedia of Genes and Genomes (KEGG) (25), protein–protein interaction (PPI), hub gene, immune-cell composition, and ROC analyses were subsequently performed. Detailed bioinformatics procedures are described in the **Supplementary Methods**.

### Patients and clinical samples

Patients diagnosed with anaphylaxis who were admitted to the Third Xiangya Hospital, Central South University, between January 2016 and October 2021 were consecutively enrolled. Eligible participants were adults aged 18–70 years. Diagnosis of anaphylaxis was established according to the EAACI guidelines (2021 update) (26). Patients with active infection, autoimmune diseases, malignant tumors, inherited coagulation disorders, or incomplete clinical records were excluded.

Peripheral venous blood samples were collected at hospital admission whenever possible before systemic treatment. Routine laboratory parameters, including neutrophil count, lymphocyte count, monocyte count, platelet count, hemoglobin, C-reactive protein, erythrocyte sedimentation rate, albumin, prothrombin time, activated partial thromboplastin time, fibrinogen, D-dimer, and fibrin degradation products (FDP), were obtained from the hospital information system. Age- and sex-matched healthy volunteers served as controls. Peripheral blood samples from healthy, age- and sex-matched volunteers and five patients with anaphylaxis were additionally collected for immunofluorescence analysis.

The study was approved by the Institutional Research Ethics Committee of the Third Xiangya Hospital, Central South University (I-22062). Written informed consent was obtained from all participants. Clinical characteristics of the enrolled patients are summarized in **Supplementary Table 1**. Detailed information regarding atopic status and serum tryptase levels was unavailable for some patients.

### Animals and experimental models

Animal experiments were approved by the Institutional Research Ethics Committee of the Third Xiangya Hospital, Central South University (CSU-2023-0208). Male BALB/c mice (6–8 weeks old) were maintained under specific pathogen-free conditions with free access to food and water. Sample sizes were selected based on previous studies using similar passive systemic anaphylaxis (PSA) and passive cutaneous anaphylaxis (PCA) models (27). Animals were randomly assigned to experimental groups using a randomization procedure. Sample sizes were selected based on previous studies using similar PSA and PCA models.

PSA and PCA models were established using anti-DNP-IgE sensitization followed by DNP-HSA challenge. PSA severity was evaluated by monitoring changes in rectal temperature after antigen challenge. PCA responses were assessed by Evans blue extravasation, ear thickness measurement, and histological analysis of ear tissues.

For pharmacological intervention experiments, mice were treated with PBS, DNase I (Roche), recombinant tissue factor pathway inhibitor (TFPI, R&D Systems), sodium heparin (Sigma, USA), or dexamethasone (DXM). DNase I was used to degrade extracellular DNA structures, whereas TFPI was used to modulate TF-related coagulation signaling. At the experimental endpoint, mice were euthanized by isoflurane overdose followed by cervical dislocation. Peripheral blood, bone marrow, and skin tissues were collected for subsequent analyses.

No animals or samples were excluded from the final analyses unless predefined experimental criteria were not met. Detailed procedures for model induction, treatment schedules, and sample collection are provided in the **Supplementary Methods**.

### Histology

Skin tissues were fixed in 10% neutral-buffered formalin, paraffin embedded, sectioned (5 μm), and stained with hematoxylin–eosin or toluidine blue. Mast cells were quantified from six randomly selected high-power fields by an investigator blinded to experimental grouping. Detailed staining procedures are described in the **Supplementary Methods**.

### Neutrophil isolation and cell culture

Human peripheral blood neutrophils and mouse bone marrow neutrophils were isolated using commercial density-gradient isolation kits (Solarbio, China) according to the manufacturer’s instructions. After isolation, neutrophils were cultured in complete IMDM medium.

For *in vitro* anaphylaxis experiments, neutrophils were pretreated with DNase I, TFPI, heparin sodium, dexamethasone, or PBS, sensitized with DNP-specific IgE for 16 hours, and subsequently stimulated with DNP-HSA for 4 hours. Detailed isolation procedures are provided in the **Supplementary Methods**.

### NET-associated extracellular DNA preparation

Human neutrophils were resuspended in RPMI 1640 (Gibco) and seeded into 24-well plates at a density of 1.8 × 10^6 cells per well. To induce extracellular DNA-associated responses, cells were stimulated with 50 nM phorbol 12-myristate 13-acetate (PMA, Sigma) for 4 hours. PMA stimulation was used to induce extracellular DNA release. After stimulation, the medium was removed, and the wells were washed with RPMI 1640. Adherent extracellular DNA structures were rinsed with 2 mL of cold PBS, and the samples were centrifuged at 1,000 g for 10 minutes at 4 °C. The supernatant was centrifuged again at 20 g for 5 minutes, and the resulting supernatant containing extracellular DNA-protein complexes was either used immediately or stored in liquid nitrogen.

### Quantification of NET-associated markers

Cell-free DNA (cfDNA) concentrations in serum and culture supernatants were determined using the Quant-iT PicoGreen dsDNA Assay (Thermo Fisher).

myeloperoxidase-DNA (MPO-DNA) complexes were quantified by capture Enzyme-Linked Immunosorbent Assay (ELISA, JianglaiBio). MPO-DNA complexes were interpreted as NET-associated biomarkers reflecting extracellular DNA release by neutrophils rather than direct evidence of NET formation. Detailed assay procedures are described in the **Supplementary Methods**.

### Immunofluorescence

Neutrophils were cultured on lysine-coated glass coverslips (Neuvitro; H-12-1.5-PDL), fixed with paraformaldehyde, blocked, and incubated with primary and fluorescent secondary antibodies. Images were acquired using a Nikon C2 confocal microscope. NET-associated extracellular structures were evaluated by co-localization of extracellular DNA with neutrophil-derived proteins including MPO, neutrophil elastase (NE), and citrullinated histone H3 (Cit-H3). Fluorescence intensity was quantified using ImageJ. Antibody information is listed in **Supplementary Table 2**, and detailed staining procedures are provided in the **Supplementary Methods**.

### Enzyme-linked immunosorbent assay (ELISA)

Blood samples were collected from patients, whereas mouse blood was collected via cardiac puncture, allowed to clot at room temperature for 30 minutes, and then centrifuged at 6000 RPM for 15 minutes at 4 °C. The plasma and supernatants were immediately stored at -80 °C. MPO-DNA complexes, NE-DNA complexes, IL-4, IL-10, TNF-α, histamine, TF, TFPI, D-dimer, and FDP levels were measured using ELISA kits (JianglaiBio) and a microplate reader (Rayto RT-6100). TF levels measured by ELISA were interpreted as TF expression rather than direct measurement of TF functional procoagulant activity.

### RNA isolation and quantitative RT-PCR

Total RNA was extracted from cultured cells using TRIzol reagent (Invitrogen) following the manufacturer’s instructions. Complementary DNA (cDNA) was synthesized using the ImProm-II Reverse Transcription System (Promega, USA). Quantitative RT-PCR was performed with SYBR Green PCR Master Mix (Applied Biosystems) on an ABI 7300 PCR system (Applied Biosystems). Gene expression levels were normalized to β-actin, and the 2^−ΔΔCt method was used for analysis. Primer sequences are detailed in **Supplementary Table 3**.

### Statistical analysis

Statistical analyses were performed using R (version 4.0.2) and GraphPad Prism (version 6). Continuous variables were tested for normality using the Shapiro–Wilk test. Normally distributed data are presented as mean ± SD, whereas non-normally distributed data are presented as median (interquartile range). Comparisons between two groups were performed using the unpaired Student’s t test or Mann–Whitney U test as appropriate. Multiple-group comparisons were analyzed using one-way ANOVA with Tukey’s *post hoc* test or the Kruskal–Wallis test with Dunn’s correction.

Repeated measurements were analyzed using two-way repeated-measures ANOVA followed by Šidák’s multiple-comparison test. Correlations were assessed using Pearson or Spearman correlation analyses according to data distribution. ROC analyses were performed using the pROC package, and the area under the curve (AUC) with 95% confidence intervals was calculated. For transcriptomic analyses, P values were adjusted using the Benjamini–Hochberg false discovery rate method.

A two-sided P < 0.05 was considered statistically significant.

## Results

### Identification and functional characterization of NET-associated genes in anaphylaxis

**Figure 1:**
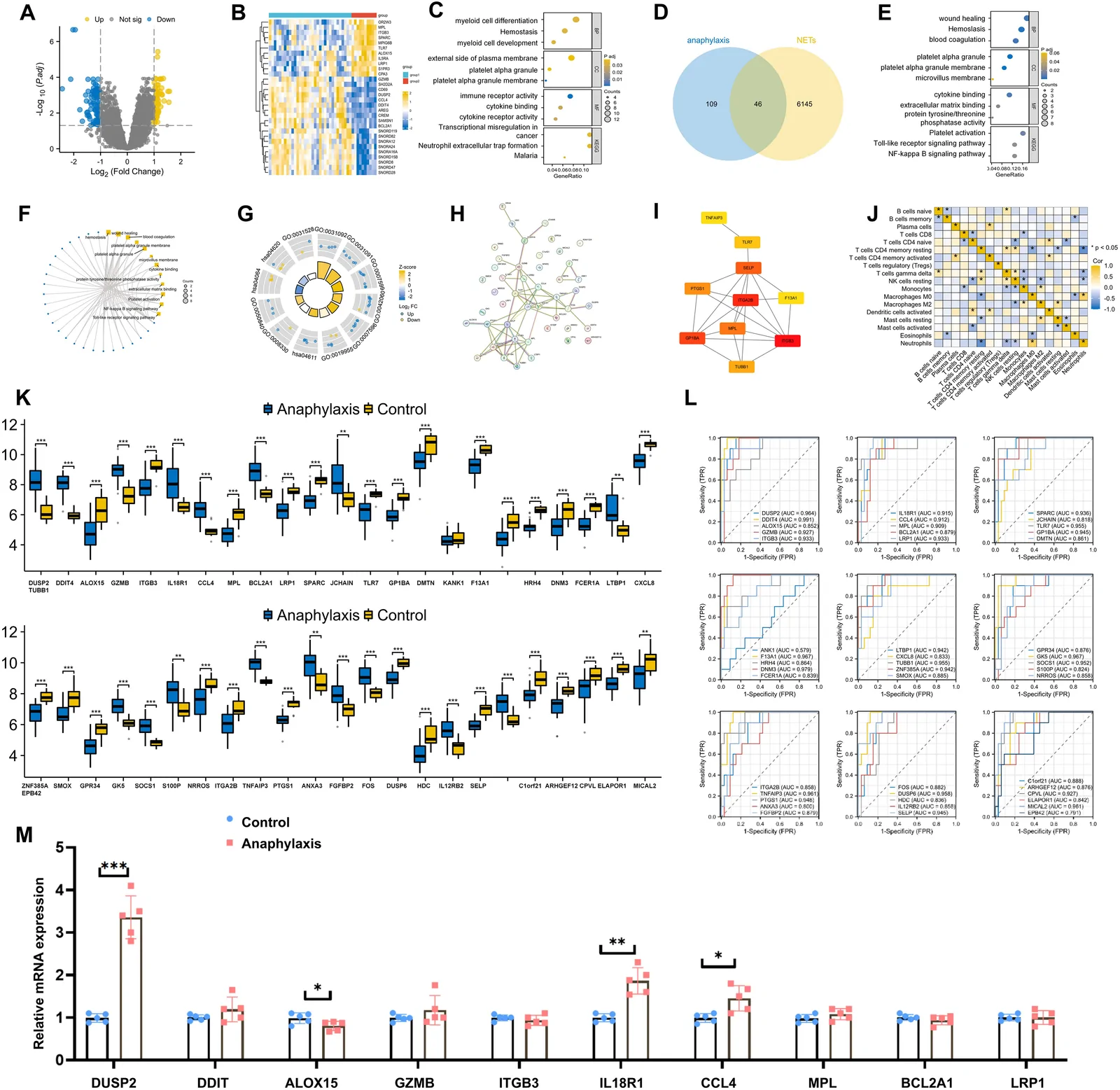
Identification and functional enrichment analysis of NET-associated genes in anaphylaxis. **(A)** The volcano plot shows the differential expression analysis results of the GSE69063 dataset. **(B)** The heatmap of differential expression analysis results from the GSE69063 dataset. **(C)** Functional enrichment analysis of the GSE69063 dataset. **(D)** Venn diagram showing the intersection of NET-associated candidate genes and GSE69063 differentially expressed genes obtained from MSigDB and/or GeneCards databases. **(E)** GO and KEGG enrichment bubble plot of NET-associated candidate genes in anaphylaxis. **(F)** Circular plot of GO and KEGG enrichment of NET-associated candidate genes in anaphylaxis. **(G)** Circos mapping of functional enrichment in the GSE69063 dataset combined with log(fold change) values. **(H)** PPI network of NET-associated candidate genes in anaphylaxis based on the STRING database. **(I)** Top 10 hub genes in the PPI network. **(J)** Heatmap of immune cell correlations in the dataset. **(K)** Box plot showing the expression levels of selected NET-associated genes in the dataset. **(L)** ROC curve. **(M)** qRT-PCR validation of differential expression of potential diagnostic markers. *P < 0.05, **P < 0.01, ***P < 0.001.

To identify NET-associated candidate genes potentially associated with anaphylaxis, transcriptomic analysis was performed using the GSE69063 dataset. Differential expression analysis identified 155 differentially expressed genes (DEGs) between patients with anaphylaxis and healthy controls, including 106 upregulated and 49 downregulated genes (**Figures 1A–C**).

NET-associated candidate genes obtained from the GeneCards and MSigDB databases were intersected with the identified DEGs. A total of 46 NET-associated DEGs were identified and selected for subsequent analyses (**Figure 1D**).

Functional enrichment analysis showed that these NET-associated DEGs were mainly related to biological processes including wound healing and blood coagulation. Enriched cellular components included platelet alpha granules, while molecular functions involved cytokine binding, extracellular matrix binding, and protein tyrosine/threonine phosphatase activity. KEGG pathway analysis indicated enrichment in pathways related to platelet activation, Toll-like receptor signaling, and NF-κB signaling (**Figures 1E–G**).

A protein-protein interaction (PPI) network was constructed to characterize interactions among NET-associated DEGs. Network analysis identified the top 10 hub genes based on network topology (**Figures 1H, I**) The relative proportions of 22 immune-cell populations were estimated using the CIBERSORT algorithm and visualized as heatmaps (**Figure 1J**). Because these results were computationally inferred from bulk transcriptomic data, they were interpreted as estimated immune-cell composition rather than direct measurements of immune-cell infiltration.

ROC curve analysis demonstrated that multiple NET-associated genes showed potential ability to distinguish anaphylaxis samples from controls (**Figures 1K, L**). To validate selected candidate genes, peripheral blood samples from five patients with anaphylaxis and five healthy controls were analyzed. Expression levels of *DUSP2*, *IL18R1*, and *CCL4* were increased, whereas *ALOX15* expression was decreased in patients with anaphylaxis (**Figure 1M**).

Together, these findings indicate that NET-associated transcriptional changes occur in anaphylaxis and are associated with inflammatory and coagulation-related biological pathways.

### NET-associated responses are increased in human and experimental models of anaphylaxis

**Figure 2:**
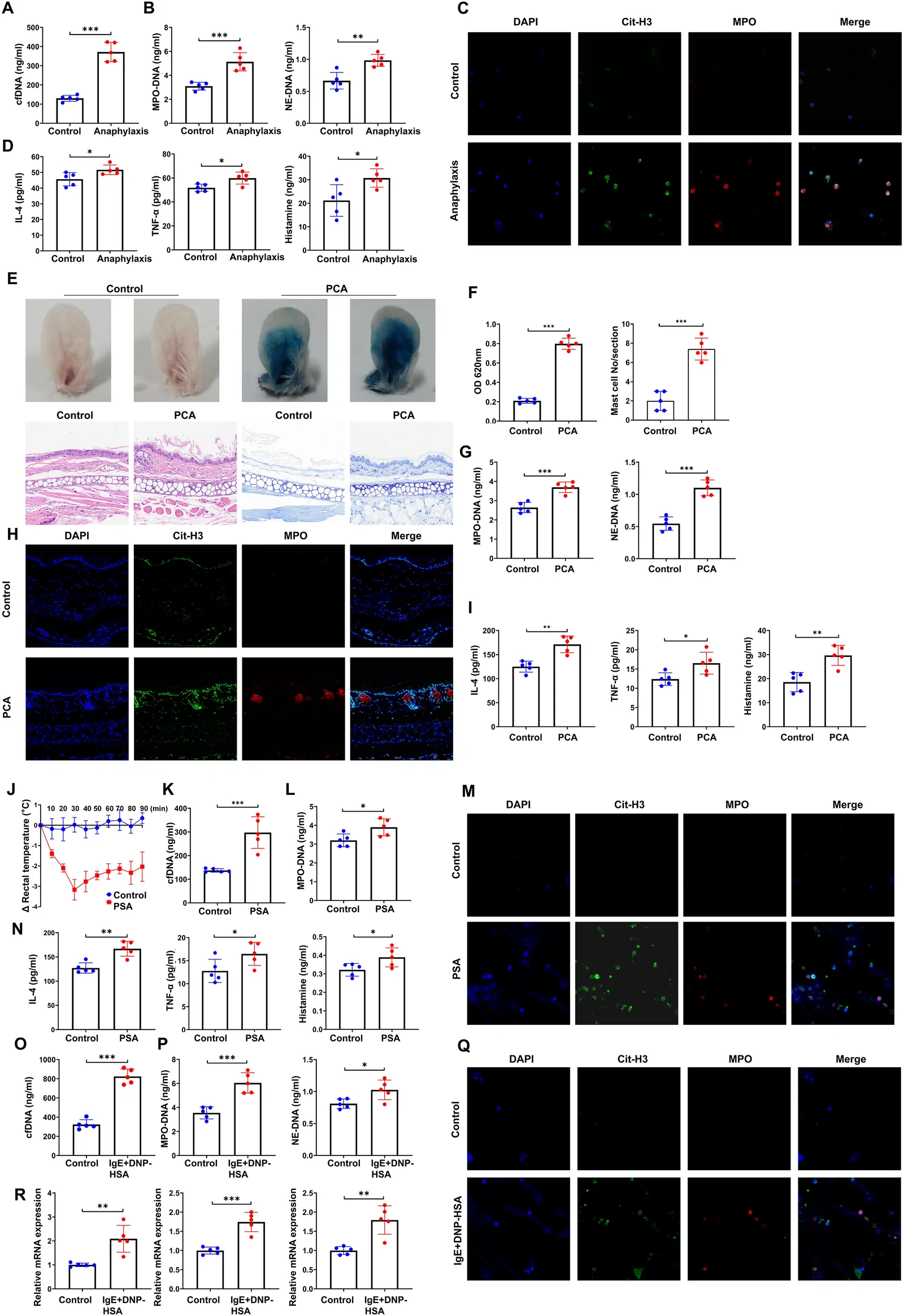
NET-associated responses are increased in human and experimental anaphylaxis. Serum levels of **(A)** cell-free DNA (cfDNA), **(B)** MPO–DNA complexes and NE–DNA complexes. **(C)** Representative immunofluorescence images showing NET-associated extracellular DNA structures in healthy controls and patients with anaphylaxis (red: MPO, green: CitH3, blue: DAPI). **(D)** Levels of IL-4, TNF-α, and histamine in the plasma of patients and healthy controls were measured by ELISA. **(E–I)**. BALB/c mice were established as a passive cutaneous anaphylaxis (PCA) model by subcutaneous injection of 1 μg anti-DNP-IgE into the right ear, followed by intravenous challenge with 100 μg DNP-HSA dissolved in saline containing 4% Evans blue. **(E)** Representative images showing Evans blue extravasation in the ears and corresponding ear tissue sections from control and PCA mice. **(F)** Quantification of Evans blue extravasation and mast cell counts in ear tissues. **(G)** Serum cfDNA levels and MPO–DNA complex levels. **(H)** Representative immunofluorescence images showing NET-associated extracellular DNA structures in control and PCA mice (red, MPO; green, Cit-H3; blue, DAPI). **(I)** Plasma IL-4, TNF-α, and histamine levels measured by ELISA. **(J–N)** BALB/c mice were sensitized with 10 μg anti-DNP-IgE and challenged intravenously with 100 μg DNP-HSA. **(J)** Rectal temperature. **(K)** Serum cfDNA levels. **(L)** MPO–DNA complexes levels. **(M)** Representative immunofluorescence images showing NET-associated extracellular DNA structures in control and PCA mice (red, MPO; green, Cit-H3; blue, DAPI). **(N)** Plasma IL-4, TNF-α, and histamine levels measured by ELISA. **(O–R)**. Primary neutrophils were sensitized with anti-DNP-IgE and treated with DNP-HSA. **(O)** Supernatant cfDNA, **(P)** MPO–DNA complexes and NE–DNA complexes. **(Q)** Representative immunofluorescence images showing NET-associated extracellular DNA structures (red, MPO; green, Cit-H3; blue, DAPI). **(R)** Relative mRNA expression of IL-4, TNF-α, and histamine. *P < 0.05, **P < 0.01, ***P < 0.001.

To explore whether transcriptomic alterations were accompanied by changes in NET-associated responses, we measured NET-associated markers in patients with anaphylaxis and healthy controls. Compared with healthy controls, patients with anaphylaxis showed increased levels of cell-free DNA(cfDNA), MPO-DNA complexes, and NE-DNA complexes (**Figures 2A, B**). Immunofluorescence analysis of peripheral blood neutrophils demonstrated increased NET-associated extracellular DNA signals co-localizing with Cit-H3 and MPO in patients with anaphylaxis compared with controls (**Figure 2C**).In addition, pro-inflammatory cytokines associated with allergic responses, including IL-4, TNF-α, and histamine were increased in anaphylaxis patients (**Figure 2D**).

We next evaluated NET-associated responses in a PCA mouse model. Compared with control mice, PCA mice showed increased Evans blue extravasation and vascular permeability, accompanied by enhanced mast-cell degranulation (**Figures 2E, F**). Increased NET-associated extracellular DNA structures co-localizing with Cit-H3 and MPO were observed in PCA skin tissues, together with elevated MPO-DNA, NE-DNA, IL-4, TNF-α, and histamine levels (**Figures 2G–I**).

These findings were further validated in a PSA mouse model, which showed increased NET-associated responses and elevated cytokines (**Figures 2J–N**). Similarly, primary neutrophils cultured from mouse bone marrow displayed similar increases in NET-associated responses and inflammatory cytokine production following stimulation with IgE and DNP-HSA (**Figures 2O–R**). Together, these results demonstrate that NET-associated responses are increased in human and experimental models of anaphylaxis.

### DNase I treatment is associated with reduced NET-associated responses and experimental anaphylaxis

**Figure 3:**
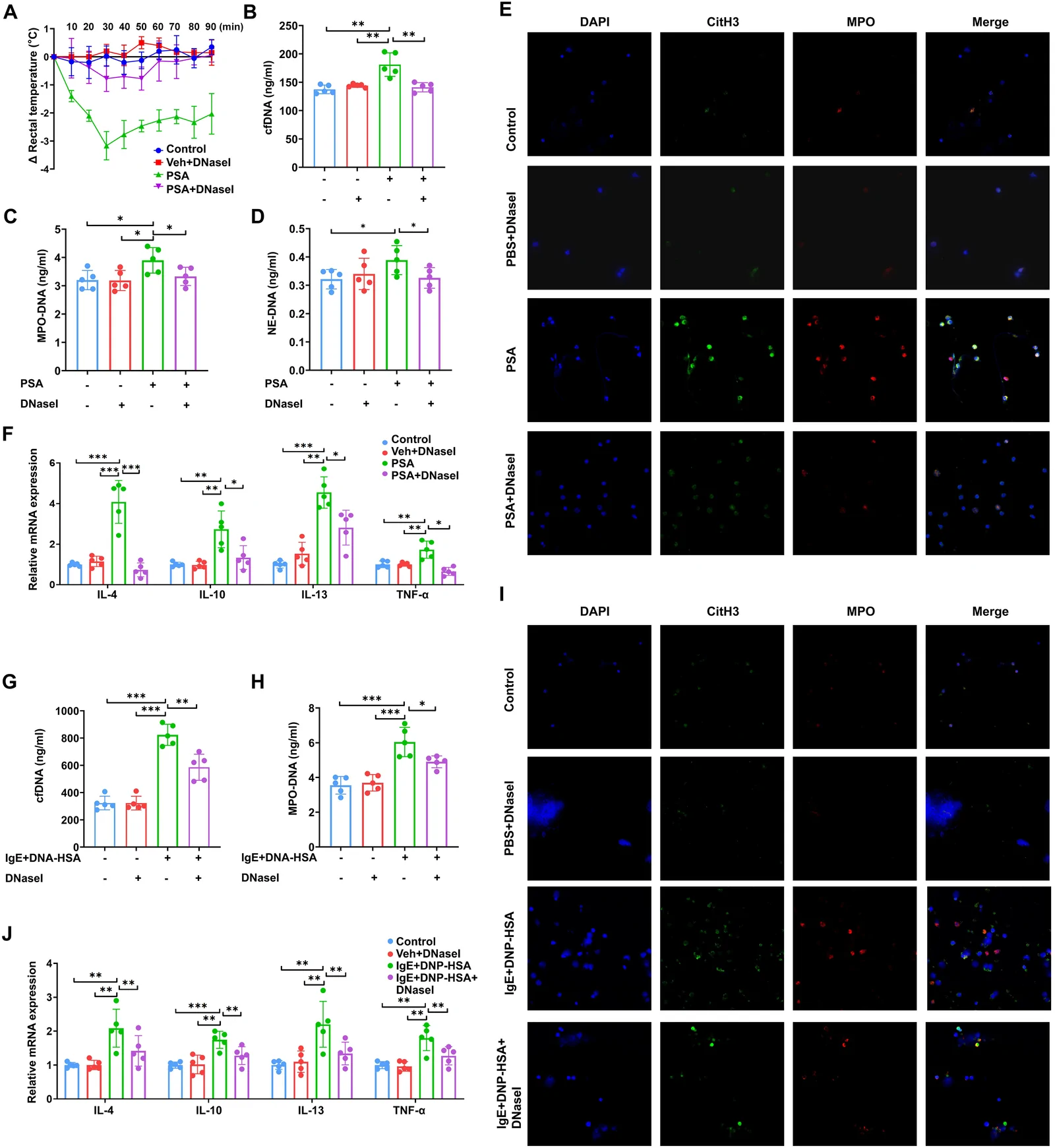
DNase I treatment is associated with reduced NET-associated responses and experimental anaphylaxis. **(A–F)**. BALB/c mice were sensitized with 10 μg anti-DNP-IgE and challenged intravenously with 100 μg DNP-HSA with or without DNase I pretreatment. **(A)** Rectal temperature. **(B)** Levels of serum cfDNA and **(C)** MPO-DNA complexes, **(D)** NE-DNA complexes. **(E)** Representative immunofluorescence images showing NET-associated extracellular DNA structures (red, MPO; green, Cit-H3; blue, DAPI). **(F)** Relative mRNA expression of IL-4, IL-10, IL-13, and TNF-α. **(G–J)** Primary neutrophils were sensitized with anti-DNP-IgE and treated with DNP-HSA with or without DNase I pretreatment. **(G)** Supernatant cfDNA. **(H)** MPO-DNA complexes. **(I)** Representative immunofluorescence images showing NET-associated extracellular DNA structures. **(J)** Relative mRNA expression of IL-4, IL-10, IL-13, and TNF-α. *P < 0.05, **P < 0.01, ***P < 0.001.

To investigate whether modulation of extracellular DNA-associated responses affects experimental anaphylactic responses, PSA mice were treated with DNase I. Compared with untreated PSA mice, DNase I-treated mice exhibited a less pronounced decrease in body temperature following antigen challenge and showed accelerated temperature recovery (**Figure 3A**). Furthermore, DNase I treatment was accompanied by reduced levels of cell-free DNA (cfDNA), MPO-DNA complexes, and NE-DNA complexes compared with untreated PSA mice (**Figures 3B–D**). Immunofluorescence analysis of peripheral blood neutrophils demonstrated decreased extracellular DNA-associated signals following DNase I treatment. In addition, the co-localization of extracellular DNA signals with Cit-H3 and MPO was reduced in DNase I-treated mice compared with PSA mice (**Figure 3E**). Furthermore, DNase I treatment was associated with reduced inflammatory cytokine levels, including IL-4, IL-10, IL-13, and TNF-α (**Figure 3F**). Similar effects were observed in IgE-stimulated primary neutrophils, in which DNase I treatment was accompanied by decreased extracellular DNA-associated markers and inflammatory cytokine production (**Figures 3G–J**). These findings suggest that degradation of extracellular DNA structures is associated with attenuation of inflammatory responses during experimental anaphylaxis.

## NET-associated responses are associated with TF expression during anaphylaxis

**Figure 4:**
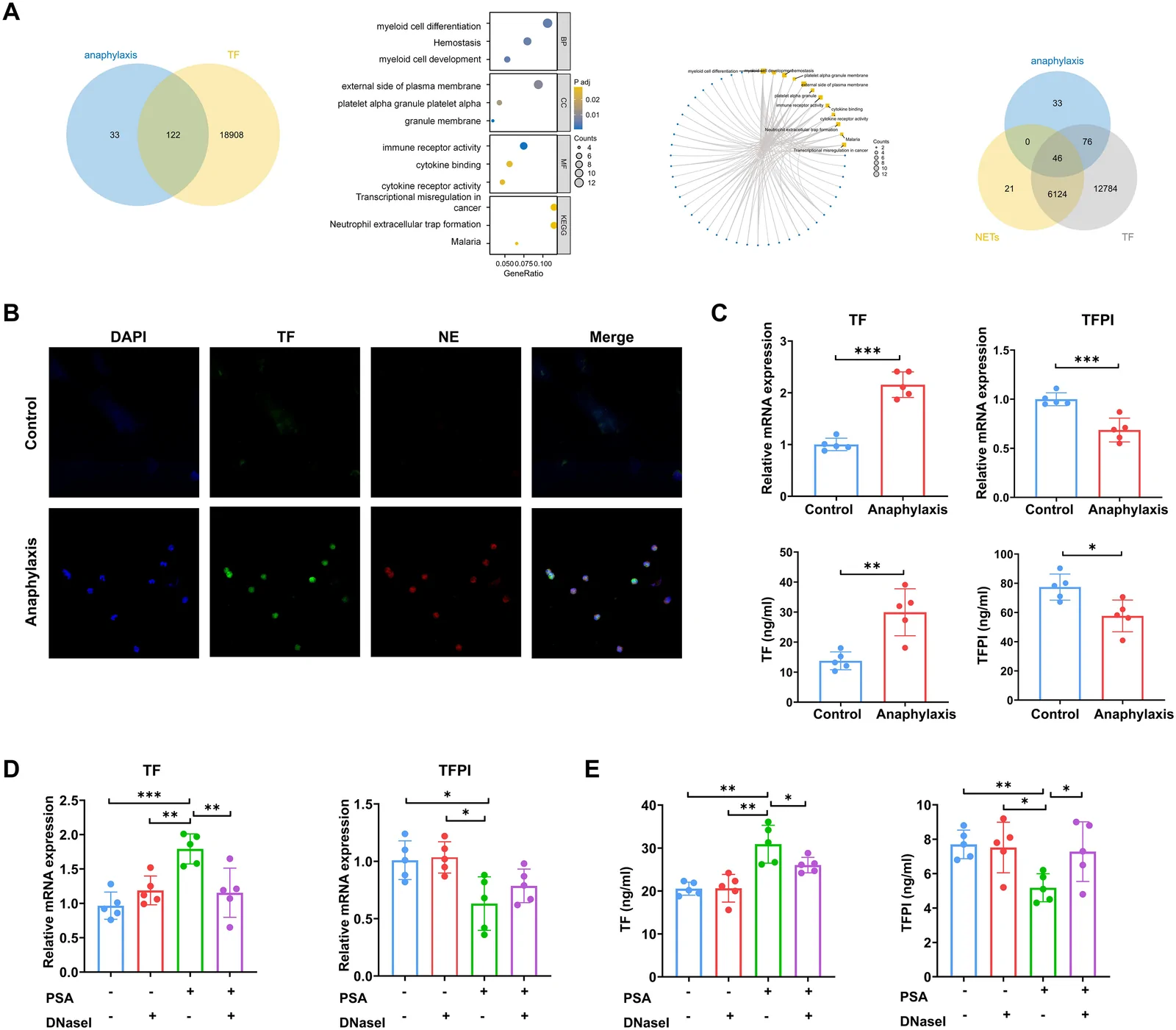
Increased tissue factor expression is associated with NET-associated responses during anaphylaxis. **(A)** Differential expression of TF-related genes together with GO and KEGG enrichment analyses of NET-associated genes in the GSE69063 dataset. **(B)** Representative immunofluorescence images showing TF expression and NE staining in healthy controls and patients with anaphylaxis (red: NE, green: TF, blue: DAPI). **(C)** Plasma TF and TFPI protein levels and corresponding mRNA expression levels were measured. **(D)** Plasma TF and TFPI levels were measured in healthy controls and PSA mice treated with saline or DNase I, and **(E)** mRNA levels were measured. *P < 0.05, **P < 0.01, ***P < 0.001.

Because NET-associated structures have been linked to thrombo-inflammatory processes, we next investigated their relationship with TF expression during anaphylaxis. Analysis of the GSE69063 dataset revealed correlations between NET-associated genes and TF expression in anaphylaxis samples (**Figure 4A**).

To evaluate the spatial relationship between TF expression signals and neutrophil-associated extracellular structures in PSA mice, bone marrow neutrophils were isolated from PSA mice and subjected to immunofluorescence analysis for TF and NE. Immunofluorescence staining demonstrated an increased spatial association between TF fluorescence signals and NE-positive structures in neutrophils from PSA mice compared with controls (**Figure 4B**). Plasma TF levels were also increased in experimental anaphylaxis (**Figure 4C**). DNase I treatment in PSA mice was accompanied by reduced NET-associated responses, decreased TF expression, and increased TFPI levels (**Figures 4D, E**). These findings indicate that NET-associated responses are associated with TF expression and coagulation-related alterations during anaphylaxis.

## TF pathway modulation is associated with reduced experimental anaphylactic responses

**Figure 5:**
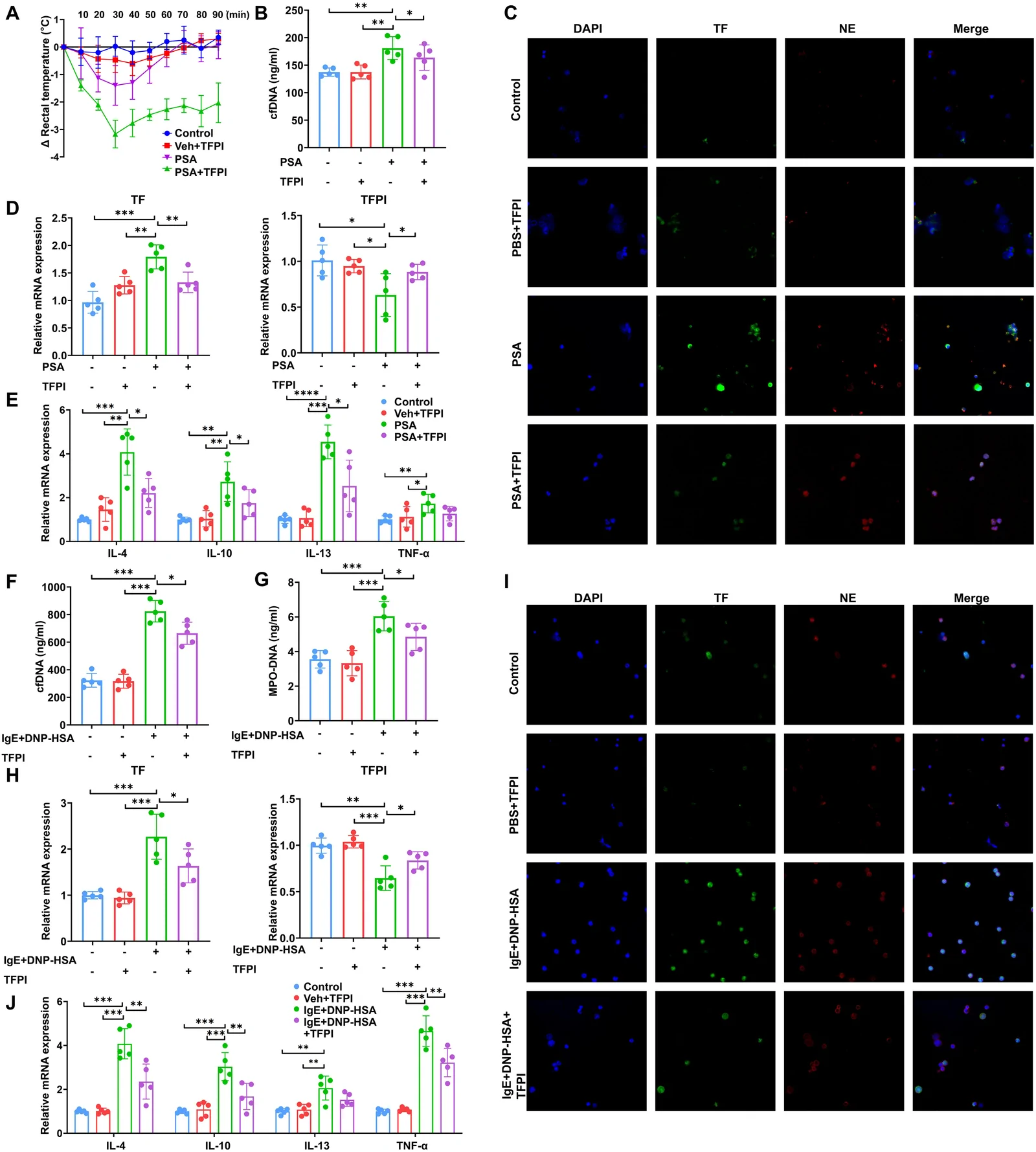
TFPI treatment Is associated with reduced NET-associated markers and inflammatory responses. **(A–E)**. BALB/c mice were sensitized with 10 μg anti-DNP-IgE and challenged intravenously with 100 μg DNP-HSA with or without TFPI pretreatment. **(A)** Rectal temperature changes in the mouse model. **(B)** Serum cfDNA levels. **(C)** Representative immunofluorescence images showing TF expression and NE staining (red: NE, green: TF, blue: DAPI). **(D)** Relative mRNA expression of TF and TFPI. **(E)** Relative mRNA expression of IL-4, IL-10, IL-13, and TNF-α. **(F–J)** Primary neutrophils were sensitized with anti-DNP-IgE and treated with DNP-HSA with or without TFPI pretreatment. **(F)** Supernatant cfDNA. **(G)** MPO-DNA complexes. **(H)** Relative TF and TFPI mRNA expression. **(I)** Representative immunofluorescence images showing TF expression and NE staining (red: NE, green: TF, blue: DAPI). **(J)** Relative mRNA expression of IL-4, IL-10, IL-13, and TNF-α. *P < 0.05, **P < 0.01, ***P < 0.001, ****P < 0.0001.

To further investigate the relationship between TF-related pathways and anaphylactic responses, PSA mice and primary neutrophils were treated with recombinant TFPI. In PSA mice, TFPI treatment was accompanied by improved temperature recovery following antigen challenge, reduced inflammatory cytokine levels, reduced TF expression levels, and lower NET-associated marker levels (**Figures 5A–E**).

These findings were further supported by experiments using IgE-stimulated primary neutrophils *in vitro* (**Figures 5F–J**). Together, these findings suggest that modulation of TF-related pathways is associated with reduced inflammatory activation and NET-associated responses during experimental anaphylaxis.

### Coagulation abnormalities are associated with anaphylaxis severity and NET-associated markers

**Figure 6:**
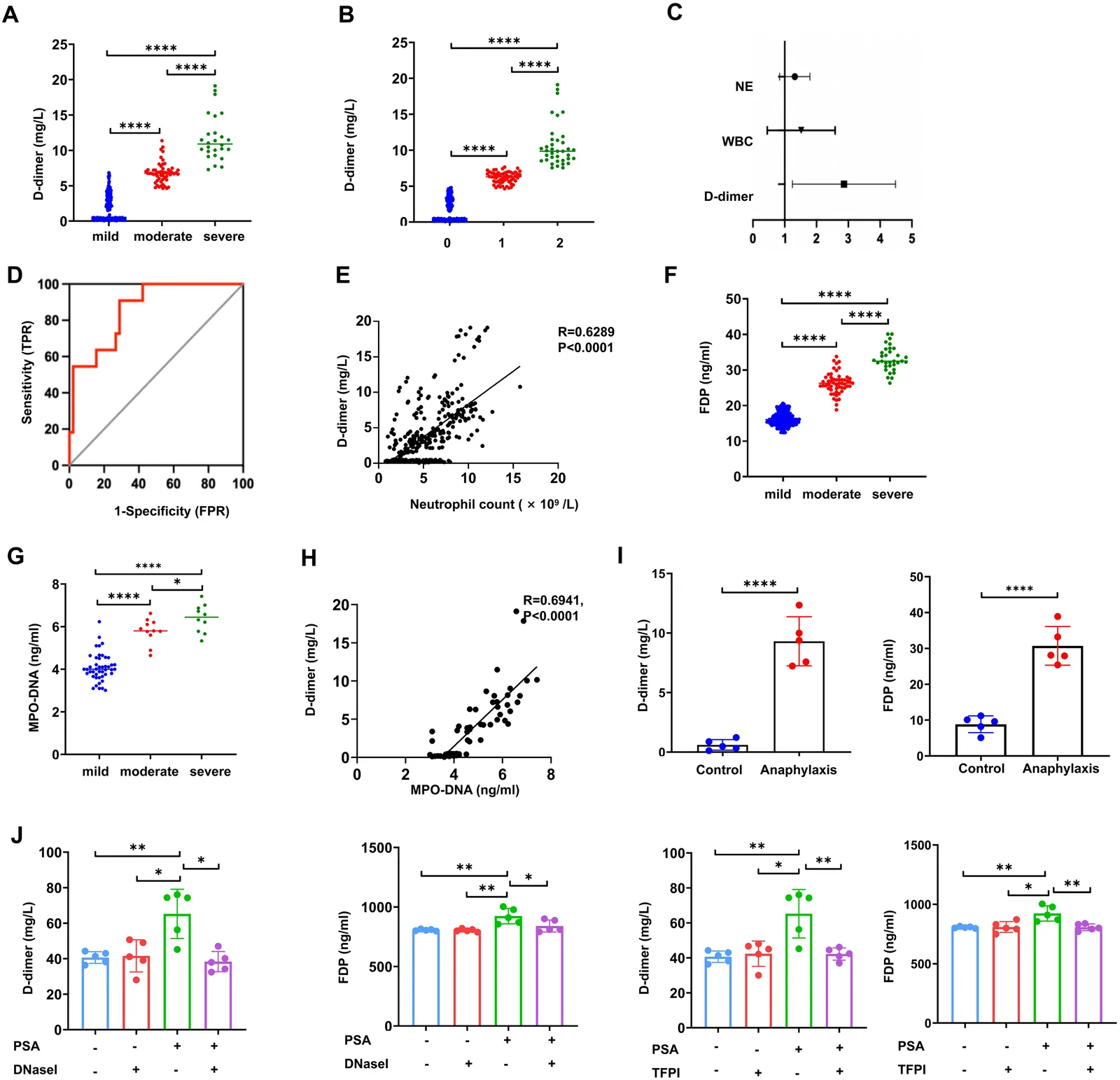
Coagulation abnormalities are associated with anaphylaxis severity and NET-associated markers. **(A)** D-dimer levels in patients with mild, moderate, and severe anaphylaxis. **(B)** D-dimer levels in anaphylaxis patients with 0, 1, and 2 instances of anaphylactic shock (AS). **(C)** Forest plot of multivariate logistic regression analysis of disease severity in anaphylaxis patients. **(D)** Receiver operating characteristic (ROC) curve evaluating the discriminatory performance of D-dimer for disease severity. **(E)** Correlation between D-dimer levels and neutrophil counts. **(F)** FDP levels in patients with mild, moderate, and severe anaphylaxis. **(G)** MPO-DNA levels in patients with different severities of anaphylaxis. **(H)** Correlation between D-dimer levels and MPO-DNA levels. **(I)** D-dimer and FDP levels in patients with anaphylaxis and healthy controls. **(J)** D-dimer and FDP levels in PSA mice with or without DNaseI, TFPI treatment. Data are presented as mean ± SD or median (interquartile range), as appropriate. *P < 0.05, **P < 0.01, ***P < 0.001, ****P < 0.0001.

Given the relationship between TF expression and coagulation pathways, we further investigated coagulation-related abnormalities in patients with anaphylaxis. Clinical data from 323 patients with anaphylactic reactions were retrospectively analyzed. Patients with more severe anaphylactic reactions exhibited higher D-dimer levels compared with patients with less severe reactions (**Figure 6A**). Elevated D-dimer levels were also associated with the occurrence of anaphylactic shock (**Figure 6B**). Multivariable regression analysis showed that increased D-dimer levels were independently associated with greater anaphylaxis severity (**Figure 6C**). ROC analysis demonstrated that D-dimer levels showed potential value in distinguishing patients with different severity categories of anaphylaxis (AUC = 0.8626; **Figure 6D**).

Correlation analysis showed that D-dimer levels were positively correlated with neutrophil counts (R = 0.6289, P < 0.0001) (**Figure 6E**). FDP levels were also associated with the severity of the anaphylactic reaction (**Figure 6F**). Among 66 patients with available MPO-DNA measurements, MPO-DNA levels differed significantly among severity groups and were positively correlated with D-dimer levels (R = 0.6941, P < 0.0001) (**Figures 6G, H**).

Furthermore, D-dimer and FDP levels were increased in patients with anaphylaxis compared with healthy controls and were elevated in PSA mice compared with control mice (**Figures 6I, J**). DNase I and TFPI treatments were associated with reduced D-dimer and FDP levels in PSA mice (**Figure 6J**). Together, these findings indicate that coagulation abnormalities are associated with anaphylaxis severity and NET-associated responses.

### Heparin sodium and dexamethasone (DXM) are associated with reduced experimental anaphylactic responses and NET-associated responses

**Figure 7:**
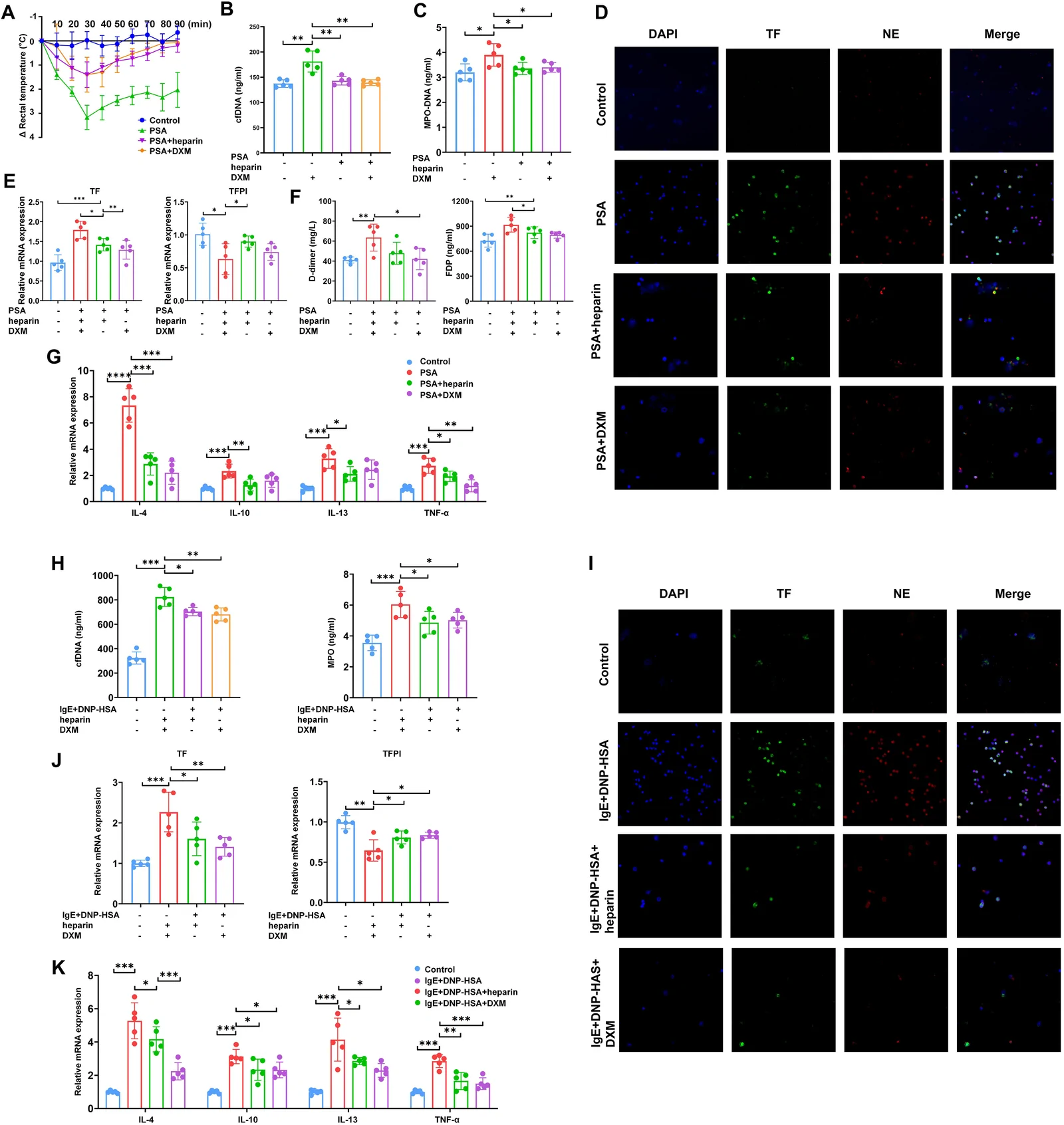
Heparin sodium and dexamethasone (DXM) are associated with reduced anaphylactic responses and NET-associated markers in experimental anaphylaxis. **(A–G)** BALB/c mice were sensitized with 10 μg anti-DNP-IgE and challenged intravenously with 100 μg DNP-HSA with or without heparin sodium or DXM treatment. **(A)** Rectal temperature. **(B)** Serum cfDNA. **(C)** MPO-DNA complexes. **(D)** Representative confocal immunofluorescence images showing TF expression and NE staining (red, NE; green, TF; blue, DAPI). **(E)** Relative TF and TFPI mRNA expression. **(F)** D-dimer and FDP levels. **(G)** Relative mRNA expression of IL-4, IL-10, IL-13, and TNF-α. **(H–K)** Primary neutrophils were sensitized with anti-DNP-IgE and treated with DNP-HSA with or without heparin sodium or DXM treatment. **(H)** Supernatant cfDNA and MPO–DNA complexes. **(I)** Representative immunofluorescence images showing TF expression and NE staining (red: NE, green: TF, blue: DAPI). **(J)** Relative TF and TFPI mRNA expression. **(K)** Relative mRNA expression of IL-4, IL-10, IL-13, and TNF-α. *P < 0.05, **P < 0.01, ***P < 0.001, ****P < 0.0001.

Based on the observed coagulation abnormalities during anaphylaxis, we further evaluated the effects of heparin sodium in experimental models, using dexamethasone (DXM) as an anti-inflammatory reference treatment. In PSA mice, both heparin sodium and DXM were associated with improved temperature recovery, reduced inflammatory cytokine levels, and decreased NET-associated markers, TF expression, D-dimer, and FDP levels (**Figures 7A–G**).

Similar effects were observed in IgE-stimulated primary neutrophils, where heparin sodium and DXM were accompanied by reductions in extracellular DNA-associated markers and inflammatory responses (**Figures 7H–K**).

These findings suggest that heparin sodium is associated with reduced experimental anaphylactic responses together with changes in coagulation-related and NET-associated markers. However, whether these effects are mediated primarily through anticoagulant activity, anti-inflammatory effects, or other mechanisms requires further investigation.

## Discussion

In this study, we found that NET-associated responses were observed together with increased TF expression and coagulation abnormalities during anaphylaxis. Our microarray analysis showed that NET-associated genes were significantly altered in anaphylaxis, and several candidate genes were differentially expressed in patients.

We detected elevated levels of NET-associated markers and TF in patients with anaphylaxis and in PCA and PSA mouse models. Treatment with DNase I, which degrades extracellular DNA structures, was associated with attenuation of experimental anaphylactic manifestations. Furthermore, TFPI treatment was accompanied by reduced inflammatory responses and improved anaphylactic phenotypes. We also observed abnormal coagulation profiles in patients with anaphylaxis, including elevated levels of D-dimer and FDP compared to healthy controls. Treatment with heparin sodium in PSA mice and in IgE-stimulated neutrophils similarly attenuated inflammatory and anaphylactic responses. These findings suggest association among NET-associated responses, TF expression, coagulation abnormalities, and anaphylaxis, and provide rationale for further investigation of coagulation-related mechanisms and potential therapeutic strategies.

Anaphylaxis is a life-threatening systemic hypersensitivity reaction characterized by rapid onset and involvement of multiple organ (26, 28). A central mechanism involves mast-cell activation and the release of preformed mediators such as histamine following FcϵRI cross-linking (29). Previous studies have suggested an important contribution of neutrophil activation to severe anaphylaxis, particularly in food-induced reactions (30, 31). In our study, increased NET-associated markers were observed in patients with anaphylaxis and in both PCA and PSA mouse models. In PCA mice, increased mast-cell degranulation was accompanied by enhanced NET-associated signals, suggesting that IgE-mediated allergic responses may involve both mast-cell activation and neutrophil-associated extracellular DNA responses. Activated neutrophils can release extracellular DNA structures decorated with histones, proteases, and other proteins. (32, 33). Accordingly, excessive NET-associated extracellular components may amplify inflammatory responses under pathological conditions.

Additionally, we evaluated the potential contribution of extracellular DNA-associated responses to experimental anaphylaxis. DNase I, which degrades extracellular DNA structures, was associated with reduced experimental anaphylactic responses in both *in vivo* and *in vitro* experiments. DNase I treatment reduced NET-associated markers and pro-inflammatory cytokine levels in murine models of allergic responses. These findings suggest that extracellular DNA-associated components may contribute to inflammatory amplification during anaphylaxis. However, because DNase I is not specific to NET formation, the observed effects cannot be attributed exclusively to NET degradation. Therefore, targeting extracellular DNA-associated inflammatory components may represent a potential strategy worthy of further investigation.

TF is a transmembrane cofactor that plays a pivotal role in initiation of the coagulation cascade (34). Dysregulated interactions between coagulation and inflammation can contribute to immunothrombosis (35, 36). Previous studies have implicated NET-associated structures in immunothrombotic processes (36), including venous thrombosis in breast and pancreatic cancers (37) and TF-associated NET structures in patients with COVID-19 (38). Coagulation factors have also been implicated in immune-mediated inflammatory diseases, including systemic vasculitis and lupus erythematosus (39). Increased TF expression has been reported in chronic spontaneous urticaria (CSU), potentially reflecting activation of coagulation pathways (40). In our study, spatial association between TF expression signals and Cit-H3-positive extracellular DNA structures in anaphylaxis, but did not establish NETs as the primary source of TF. TFPI treatment was associated with reduced TF expression, NET-associated markers, inflammatory cytokines, and anaphylactic manifestations. These findings are consistent with an association between NET-associated responses and TF-related coagulation signaling during anaphylaxis, although the direction and mechanism of this relationship require further investigation.

D-dimer is a fibrin degradation product generated during fibrinolysis (41), while FDPs comprise fragments produced during fibrin and fibrinogen breakdown (42). Elevated D-dimer levels may reflect increased coagulation and fibrinolytic activity and are widely used as markers of thrombotic activation (43, 44). D-dimer has also been associated with inflammatory responses and is elevated in several inflammatory conditions, including IgA vasculitis (45), severe pneumonia (46), COVID-19 (47), and CSU (48). In our cohort of 323 patients, D-dimer and FDP levels increased with the severity of anaphylactic reactions, and D-dimer levels were positively correlated with neutrophil counts and MPO-DNA levels. DNase I and TFPI treatment were accompanied by reductions in D-dimer and FDP levels in PSA mice, further supporting an association among extracellular DNA-associated responses, TF expression, and coagulation abnormalities. However, these findings do not establish that NETs directly mediate thrombosis during anaphylaxis. Further studies assessing TF activity and direct thrombus formation are needed to clarify this mechanism.

Heparin is used for its anticoagulant properties and also exhibits anti-inflammatory effects (49). Previous studies have evaluated inhaled heparin in inflammatory airway diseases such as asthma and chronic obstructive pulmonary disease (COPD) (50). Heparin may additionally influence oxidative and inflammatory pathways (51). Glucocorticoids have broad anti-inflammatory and immunosuppressive effects and were used in our experiments as a positive control. Based on the coagulation abnormalities observed in patients with anaphylaxis, we evaluated the effects of heparin sodium and DXM in experimental models. Both treatments attenuated anaphylactic manifestations and were accompanied by reductions in inflammatory cytokines, NET-associated markers, TF expression, D-dimer, and FDP. These findings suggest that heparin may exert beneficial effects in experimental anaphylaxis; however, whether these effects are mediated primarily through anticoagulation, anti-inflammatory activity, or other mechanisms remains unclear.

Our study has several strengths. First, we combined transcriptomic analysis with experimental validation to investigate NET-associated responses in anaphylaxis. Second, data from human samples, mouse models, and *in vitro* experiments provided complementary evidence for the association between NET-associated responses and allergic inflammation. Furthermore, multiple approaches were used to evaluate extracellular DNA- and NET-associated markers *in vivo* and *in vitro*.

However, several limitations should be acknowledged. First, the sample size of some clinical validation analyses was relatively limited, and neutrophil levels in blood and bone marrow were not systematically quantified. Second, cfDNA, MPO-DNA, NE-DNA, and Cit-H3/MPO staining are NET-associated indicators but are not individually specific for definitive NET formation. Third, DNase I primarily degrades extracellular DNA rather than specifically inhibiting NET formation, and TFPI modulates TF-related coagulation signaling but does not directly assess or selectively block TF activity. Therefore, potential off-target pharmacological effects cannot be excluded. Fourth, we did not directly assess TF procoagulant activity or histologically confirm thrombus formation. Fifth, the clinical analyses were retrospective and observational in nature and therefore cannot establish causal relationships among NET-associated responses, TF expression, and coagulation abnormalities. In addition, detailed information regarding atopic status and serum tryptase levels was not consistently available, limiting further clinical stratification. Sixth, no formal sample-size calculation was performed; experimental sample sizes were selected based on previous studies using similar PSA and PCA models. Finally, the therapeutic effects of DNase I, TFPI, or heparin were evaluated in experimental models rather than in patients, and prospective clinical studies are needed to determine their translational relevance.

## Conclusions

In summary, our findings indicate that NET-associated responses, TF expression, and coagulation abnormalities are associated with anaphylaxis severity and experimental disease manifestations. Increased NET-associated markers, TF expression, and coagulation-related indices were observed in patients with anaphylaxis and experimental models, while modulation of extracellular DNA-associated responses and TF-related pathways was accompanied by reduced inflammatory responses and experimental anaphylactic manifestations. These findings support a potential association among NET-associated responses, TF expression, and coagulation abnormalities during anaphylaxis, rather than establishing a definitive causal pathway. Further studies assessing TF activity, coagulation function, and specific NET formation are required to clarify the underlying mechanisms and therapeutic relevance.

## Data Availability

The original contributions presented in the study are included in the article/**Supplementary Material**. Further inquiries can be directed to the corresponding author.

## References

[1] Turner PJ Campbell DE Motosue MS Campbell RL . Global trends in anaphylaxis epidemiology and clinical implications. J Allergy Clin Immunol In Pract. (2020) 8:1169–76. doi: 10.1016/j.jaip.2019.11.027 31786255 PMC7152797

[2] Asai Y Yanishevsky Y Clarke A La Vieille S Delaney JS Alizadehfar R . Rate, triggers, severity and management of anaphylaxis in adults treated in a Canadian emergency department. Int Arch Allergy Immunol. (2014) 164:246–52. doi: 10.1159/000365631 25170673

[3] Ma L Danoff TM Borish L . Case fatality and population mortality associated with anaphylaxis in the United States. J Allergy Clin Immunol. (2014) 133:1075–83. doi: 10.1016/j.jaci.2013.10.029 24332862 PMC3972293

[4] Motosue MS Bellolio MF Van Houten HK Shah ND Campbell RL . Increasing emergency department visits for anaphylaxis, 2005-2014. J Allergy Clin Immunol In Pract. (2017) 5:171–175.e173. doi: 10.1016/j.jaip.2016.08.013 27818135

[5] Turner PJ Gowland MH Sharma V Ierodiakonou D Harper N Garcez T . Increase in anaphylaxis-related hospitalizations but no increase in fatalities: an analysis of United Kingdom national anaphylaxis data, 1992-2012. J Allergy Clin Immunol. (2015) 135:956–963.e951. doi: 10.1016/j.jaci.2014.10.021 25468198 PMC4382330

[6] Reber LL Hernandez JD Galli SJ . The pathophysiology of anaphylaxis. J Allergy Clin Immunol. (2017) 140:335–48. doi: 10.1016/j.jaci.2017.06.003 28780941 PMC5657389

[7] Asero R Tedeschi A Coppola R Griffini S Paparella P Riboldi P . Activation of the tissue factor pathway of blood coagulation in patients with chronic urticaria. J Allergy Clin Immunol. (2007) 119:705–10. doi: 10.1016/j.jaci.2006.08.043 17204316

[8] Park J Wysocki RW Amoozgar Z Maiorino L Fein MR Jorns J . Cancer cells induce metastasis-supporting neutrophil extracellular DNA traps. Sci Transl Med. (2016) 8:361ra138. doi: 10.1126/scitranslmed.aag1711 27798263 PMC5550900

[9] Francis A Bosio E Stone SF Fatovich DM Arendts G MacDonald SPJ . Markers involved in innate immunity and neutrophil activation are elevated during acute human anaphylaxis: validation of a microarray study. J Innate Immun. (2019) 11:63–73. doi: 10.1159/000492301 30189430 PMC6738247

[10] McGrath FM Francis A Fatovich DM Macdonald SP Arendts G Woo AJ . Genes involved in platelet aggregation and activation are downregulated during acute anaphylaxis in humans. Clin Trans Immunol. (2022) 11:e1435. doi: 10.1002/cti2.1435 36583159 PMC9791329

[11] Kim TS Silva LM Theofilou VI Greenwell-Wild T Li L Williams DW . Neutrophil extracellular traps and extracellular histones potentiate IL-17 inflammation in periodontitis. J Exp Med. (2023) 220. doi: 10.1084/jem.20221751 37261457 PMC10236943

[12] Josefs T Barrett TJ Brown EJ Quezada A Wu X Voisin M . Neutrophil extracellular traps promote macrophage inflammation and impair atherosclerosis resolution in diabetic mice. JCI Insight. (2020) 5. doi: 10.1172/jci.insight.134796 32191637 PMC7205252

[13] Cui Y Yang Y Tao W Peng W Luo D Zhao N . Neutrophil extracellular traps induce alveolar macrophage pyroptosis by regulating NLRP3 deubiquitination, aggravating the development of septic lung injury. J Inflammation Res. (2023) 16:861–77. doi: 10.2147/jir.s366436 36876152 PMC9983334

[14] Hu Q Shi H Zeng T Liu H Su Y Cheng X . Increased neutrophil extracellular traps activate NLRP3 and inflammatory macrophages in adult-onset Still's disease. Arthritis Res Ther. (2019) 21:9. doi: 10.1186/s13075-018-1800-z 30616678 PMC6323819

[15] Nomura K Miyashita T Yamamoto Y Munesue S Harashima A Takayama H . Citrullinated histone H3: early biomarker of neutrophil extracellular traps in septic liver damage. J Surg Res. (2019) 234:132–8. doi: 10.1016/j.jss.2018.08.014 30527465

[16] Janssen P Tosi I Hego A Maréchal P Marichal T Radermecker C . Neutrophil extracellular traps are found in bronchoalveolar lavage fluids of horses with severe asthma and correlate with asthma severity. Front Immunol. (2022) 13:921077. doi: 10.3389/fimmu.2022.921077 35911691 PMC9326094

[17] Zhao J Jiang T Li P Dai L Shi G Jing X . Tissue factor promotes airway pathological features through epithelial-mesenchymal transition of bronchial epithelial cells in mice with house dust mite-induced asthma. Int Immunopharmacol. (2021) 97:107690. doi: 10.1016/j.intimp.2021.107690 33940323

[18] Saito R Yanase Y Kamegashira A Takayagi S Tanaka A Uchida K . Increase of tissue factor expression on the surface of peripheral monocytes of patients with chronic spontaneous urticaria. Allergy. (2020) 75:971–4. doi: 10.1111/all.14110 31715000

[19] Stakos D Skendros P Konstantinides S Ritis K . Traps N' clots: NET-mediated thrombosis and related diseases. Thromb Haemostasis. (2020) 120:373–83. doi: 10.1055/s-0039-3402731 31940675

[20] Barrett T Troup DB Wilhite SE Ledoux P Evangelista C Kim IF . NCBI GEO: archive for functional genomics data sets--10 years on. Nucleic Acids Res. (2011) 39:D1005–1010. doi: 10.1093/nar/gkq1184 21097893 PMC3013736

[21] Davis S Meltzer PS . GEOquery: a bridge between the gene expression omnibus (GEO) and bioConductor. Bioinf (Oxford England). (2007) 23:1846–7. doi: 10.1093/bioinformatics/btm254 17496320

[22] Stelzer G Rosen N Plaschkes I Zimmerman S Twik M Fishilevich S . The GeneCards Suite: from gene data mining to disease genome sequence analyses. Curr Protoc Bioinf. (2016) 54:1.30.31–31.30.33. doi: 10.1002/cpbi.5 27322403

[23] Liberzon A Birger C Thorvaldsdóttir H Ghandi M Mesirov JP Tamayo P . The Molecular Signatures Database (MSigDB) hallmark gene set collection. Cell Syst. (2015) 1:417–25. doi: 10.1016/j.cels.2015.12.004 26771021 PMC4707969

[24] Consortium TGO . Gene Ontology Consortium: going forward. Nucleic Acids Res. (2015) 43:D1049–1056. doi: 10.1093/nar/gku1179 25428369 PMC4383973

[25] Ogata H Goto S Sato K Fujibuchi W Bono H Kanehisa M . KEGG: kyoto encyclopedia of genes and genomes. Nucleic Acids Res. (1999) 27:29–34. doi: 10.1093/nar/27.1.29 9847135 PMC148090

[26] Muraro A Worm M Alviani C Cardona V DunnGalvin A Garvey LH . EAACI guidelines: anaphylaxis (2021 update). Allergy. (2022) 77:357–77. doi: 10.1111/all.15032 34343358

[27] Kim HW Ryoo GH Jang HY Rah SY Lee DH Kim DK . NAD(+)-boosting molecules suppress mast cell degranulation and anaphylactic responses in mice. Theranostics. (2022) 12:3316–28. doi: 10.7150/thno.69684 35547746 PMC9065190

[28] Dribin TE Muraro A Camargo CA Jr. Turner PJ Wang J Roberts G . Anaphylaxis definition, overview, and clinical support tool: 2024 consensus report-a GA(2)LEN project. J Allergy Clin Immunol. (2025) 156(2):406–17.e406. doi: 10.1016/j.iac.2026.01.008 39880313 PMC12301991

[29] Gülen T Akin C . Anaphylaxis and mast cell disorders. Immunol Allergy Clinics North America. (2022) 42:45–63. doi: 10.1016/j.iac.2021.09.007 34823750

[30] Crespo JF Cabanillas B . Recent advances in cellular and molecular mechanisms of IgE-mediated food allergy. Food Chem. (2023) 411:135500. doi: 10.1016/j.foodchem.2023.135500 36682170

[31] Do AN Watson CT Cohain AT Griffin RS Grishin A Wood RA . Dual transcriptomic and epigenomic study of reaction severity in peanut-allergic children. J Allergy Clin Immunol. (2020) 145:1219–30. doi: 10.1016/j.jaci.2019.10.040 31838046 PMC7192362

[32] Jiménez-Saiz R . Drug-induced IgG-neutrophil-mediated anaphylaxis in humans: uncovered! Allergy. (2020) 75:484–5. doi: 10.1111/all.14118 31736087

[33] Papayannopoulos V . Neutrophil extracellular traps in immunity and disease. Nat Rev Immunol. (2018) 18:134–47. doi: 10.1038/nri.2017.105 28990587

[34] Mackman N . Role of tissue factor in hemostasis, thrombosis, and vascular development. Arteriosclerosis Thrombosis Vasc Biol. (2004) 24:1015–22. doi: 10.1161/01.atv.0000130465.23430.74 15117736

[35] Engelmann B Massberg S . Thrombosis as an intravascular effector of innate immunity. Nat Rev Immunol. (2013) 13:34–45. doi: 10.1038/nri3345 23222502

[36] Perdomo J Leung HHL . Immune thrombosis: exploring the significance of immune complexes and NETosis. Biology. (2023) 12. doi: 10.3390/biology12101332 37887042 PMC10604267

[37] Thålin C Hisada Y Lundström S Mackman N Wallén H . Neutrophil extracellular traps: villains and targets in arterial, venous, and cancer-associated thrombosis. Arteriosclerosis Thrombosis Vasc Biol. (2019) 39:1724–38. doi: 10.1161/atvbaha.119.312463 PMC6703916 31315434

[38] Skendros P Mitsios A Chrysanthopoulou A Mastellos DC Metallidis S Rafailidis P . Complement and tissue factor-enriched neutrophil extracellular traps are key drivers in COVID-19 immunothrombosis. J Clin Invest. (2020) 130:6151–7. doi: 10.1101/2020.06.15.20131029 32759504 PMC7598040

[39] Li S Zhou C Li W Kang L Mu H . The effects of coagulation factors on the risk of autoimmune diseases: a Mendelian randomization study. Medicine. (2024) 103:e40893. doi: 10.1097/md.0000000000040893 39969330 PMC11688059

[40] Yanase Y Takahagi S Hide M . Chronic spontaneous urticaria and the extrinsic coagulation system. Allergology Int. (2018) 67:191–4. doi: 10.1016/j.alit.2017.09.003 28993062

[41] Weitz JI Fredenburgh JC Eikelboom JW . A test in context: D-dimer. J Am Coll Cardiol. (2017) 70:2411–20. doi: 10.1016/j.jacc.2017.09.024 29096812

[42] Franchini M Focosi D Pezzo MP Mannucci PM . How we manage a high D-dimer. Haematologica. (2024) 109:1035–45. doi: 10.3324/haematol.2023.283966 37881856 PMC10985443

[43] Anderson DR Stock W Karrison TG Leader A . D-dimer and risk for thrombosis in adults with newly diagnosed acute lymphoblastic leukemia. Blood Adv. (2022) 6:5146–51. doi: 10.1182/bloodadvances.2022007699 35728059 PMC9631615

[44] Chopard R Albertsen IE Piazza G . Diagnosis and treatment of lower extremity venous thromboembolism: a review. JAMA. (2020) 324:1765–76. doi: 10.1001/jama.2020.17272 33141212

[45] Liu L Liu H Zhu K Zhang L Yin X Han L . Proteome analysis reveals novel serum biomarkers for Henoch-Schönlein purpura in Chinese children. J Proteomics. (2023) 276:104841. doi: 10.1016/j.jprot.2023.104841 36796721

[46] Zhang YX Li Y Wang Y Ren YF Yang Y Qi J . Prospective cohort study on the clinical significance of interferon-γ, D-dimer, LDH, and CRP tests in children with severe mycoplasma pneumonia. Medicine. (2024) 103:e39665. doi: 10.1097/md.0000000000039665 39465799 PMC11479529

[47] Oudkerk M Büller HR Kuijpers D Oudkerk SF van Beek EJR . d-dimer and COVID-19. Radiology. (2020) 297:E343–4. doi: 10.1148/radiol.2020203481 PMC7556118 33048037

[48] Fok JS Kolkhir P Church MK Maurer M . Predictors of treatment response in chronic spontaneous urticaria. Allergy. (2021) 76:2965–81. doi: 10.1111/all.14757 33539587

[49] Wang P Chi L Zhang Z Zhao H Zhang F Linhardt RJ . Heparin: an old drug for new clinical applications. Carbohydr Polym. (2022) 295:119818. doi: 10.1016/j.carbpol.2022.119818 35989029

[50] Shute JK Puxeddu E Calzetta L . Therapeutic use of heparin and derivatives beyond anticoagulation in patients with bronchial asthma or COPD. Curr Opin Pharmacol. (2018) 40:39–45. doi: 10.1016/j.coph.2018.01.006 29455115

[51] Potje SR Costa TJ Fraga-Silva TFC Martins RB Benatti MN Almado CEL . Heparin prevents *in vitro* glycocalyx shedding induced by plasma from COVID-19 patients. Life Sci. (2021) 276:119376. doi: 10.1016/j.lfs.2021.119376 33781826 PMC7997864

