# Supplementary material for: Neutrophil extracellular trap-associated responses are associated with tissue factor expression and coagulation abnormalities during anaphylaxis
Source: Front Immunol. 2026 Aug 25;17:1905112. doi: 10.3389/fimmu.2026.1905112 (PMC13549926; doi:10.3389/fimmu.2026.1905112)
Supplement: Supplementary file 1 [file SupplementaryFile1.docx]

**Supplementary Materials for: Neutrophil extracellular trap-associated responses are associated with tissue factor expression and coagulation abnormalities during anaphylaxis**

**Authors:** Yuzhou Huang^1,2^, Shenming Xu^1,2^, Pengfei Rong^3^, Dan wang^1,2*^, Jianyun Lu^1,2*^

**Supplementary methods**

**Supplementary Tables:** 3

**Supplementary Table 1. Clinical characteristics of 323 patients with anaphylaxis.**

**Supplementary Table 2. Information of antibodies.**

**Supplementary Table 3. Sequence of Primers and real-time RT-PCR conditions.**

# Supplementary Methods

## Bioinformatics Analysis of NET-associated Genes

### Microarray Dataset Processing and Differential Expression Analysis

The microarray dataset GSE69063 was obtained from the Gene Expression Omnibus (GEO) database (<https://www.ncbi.nlm.nih.gov/geo/>) using the GEOquery package in R.

The dataset included peripheral blood samples collected from patients with severe and moderate anaphylaxis and age- and sex-matched healthy controls. Samples were collected at admission (T0) and 1 hour after admission (T1).

Raw expression data were normalized according to the original dataset processing procedures. Differential expression analysis between anaphylaxis and control groups was performed using the limma package. Differentially expressed genes (DEGs) were identified using the criteria of absolute log₂ fold change (|log₂FC|) > 1 and adjusted P value <0.05 after Benjamini–Hochberg false discovery rate (FDR) correction.

Genes with log₂FC >1 were considered upregulated, whereas genes with log₂FC <−1 were considered downregulated. Volcano plots and heatmaps were generated using the ggplot2 and pheatmap packages.

## Identification of NET-associated Candidate Genes

NET-associated gene sets were collected from the GeneCards database (<https://www.genecards.org/>) and the Molecular Signatures Database (MSigDB) using the search term “neutrophil extracellular traps”.

To improve specificity, only genes present in both databases were retained as NET-associated candidate genes. These candidate genes were intersected with DEGs identified from GSE69063 to obtain NET-associated candidate DEGs in anaphylaxis.

## Functional Enrichment Analysis

Gene Ontology (GO) enrichment analysis and Kyoto Encyclopedia of Genes and Genomes (KEGG) pathway analysis were performed using the clusterProfiler package in R. GO enrichment included biological process (BP), cellular component (CC), and molecular function (MF) categories. Statistical significance was determined using Benjamini–Hochberg adjusted P values, with FDR <0.05 considered significant.

## Protein–Protein Interaction Network and Hub Gene Identification

Protein–protein interaction (PPI) networks were constructed using the STRING database (<https://cn.string-db.org/>) with a minimum interaction confidence score of 0.4. The interaction network was imported into Cytoscape software for visualization.

Hub genes were identified using the cytoHubba plugin based on the Maximum Clique Centrality (MCC) algorithm. The top 10 ranked genes were selected as candidate hub genes for further analysis.

## Immune Cell Composition Analysis

The CIBERSORT algorithm was applied to estimate immune-cell composition from bulk transcriptomic data using the LM22 signature matrix.

Only samples with CIBERSORT output P values <0.05 were included in downstream analyses. The relative proportions of 22 immune-cell populations were visualized using heatmaps and bar plots generated with the ggplot2 package. Because CIBERSORT estimates immune-cell composition based on transcriptional signatures, these results were interpreted as computationally inferred immune-cell proportions rather than direct measurements of immune-cell infiltration.

## ROC Curve Analysis

Receiver operating characteristic (ROC) curve analysis was performed using the pROC package in R to evaluate the ability of candidate NET-associated genes to distinguish anaphylaxis samples from healthy controls. The area under the curve (AUC) and 95% confidence intervals (CI) were calculated.

The optimal cutoff value was determined using the Youden index when applicable.

**Detailed Animal Experimental Procedures**

**Passive Systemic Anaphylaxis Model**

Passive systemic anaphylaxis (PSA) was induced as previously described. BALB/c mice received intravenous injection of 10 μg anti-DNP-IgE. After 24 hours of sensitization, mice were challenged intravenously with 100 μg DNP-HSA to induce systemic anaphylactic responses. Rectal temperature was continuously monitored every 10 minutes for 90 minutes after antigen challenge.

Blood samples were collected at the experimental endpoint for subsequent measurement of NET-associated markers, inflammatory mediators, TF-related parameters, and coagulation-related indicators.

**Passive Cutaneous Anaphylaxis Model**

For the passive cutaneous anaphylaxis (PCA) model, 1 μg anti-DNP-IgE dissolved in 20 μL PBS was injected intradermally into the right ear, while an equal volume of PBS was injected into the left ear as a control. After 24 hours, mice were challenged intravenously with 100 μg DNP-HSA dissolved in saline containing 4% Evans blue.

Thirty minutes after antigen challenge, representative images of the ears were acquired, and ear thickness was measured. Mice were subsequently euthanized, and ear tissues were collected for Evans blue quantification and histological analysis.

Evans blue dye was extracted by incubating ear tissues in 700 μL formamide for 24 hours at 63°C. Absorbance was measured at 620 nm using a spectrophotometer to quantify vascular permeability.

**Pharmacological Intervention in Animal Models**

For intervention experiments, mice were randomly divided into experimental groups (n = 5 per group). Mice received PBS, DNase I (65 U, Roche), recombinant TFPI (100 nM, R&D Systems), sodium heparin (2 IU, Sigma, USA), or dexamethasone (DXM; 1 mg/kg).

DNase I treatment was used to degrade extracellular DNA-associated structures. TFPI treatment was used to modulate TF-related coagulation signaling. Sodium heparin and DXM were used as anticoagulant and anti-inflammatory reference treatments, respectively.

The indicated treatments were administered intraperitoneally once daily for three consecutive days before antigen challenge.

**Tissue and Sample Collection**

At the experimental endpoint, mice were euthanized with isoflurane overdose followed by cervical dislocation. Peripheral blood, bone marrow, and ear skin tissues were collected.

Blood samples were processed for plasma preparation and subsequent measurement of NET-associated markers, inflammatory mediators, TF-related proteins, and coagulation parameters. Bone marrow neutrophils were isolated for downstream molecular and immunofluorescence analyses. Ear tissues from PCA experiments were collected for histological staining and Evans blue quantification.

# Neutrophil Isolation

## Human Neutrophil Isolation

Peripheral blood samples collected in EDTA-containing tubes were used for neutrophil isolation. Human peripheral blood neutrophils were isolated using the Human Peripheral Blood Neutrophil Isolation Kit (P9040, Solarbio, China) according to the manufacturer’s instructions. Briefly, fresh anticoagulated blood samples were processed within 2 h after collection whenever possible. Whole blood was carefully layered onto the density-gradient separation medium provided with the kit in sterile 15 mL centrifuge tubes to establish a discontinuous gradient. Samples were centrifuged at 600 × g for 25–30 min at room temperature without brake.

After centrifugation, the granulocyte layer was carefully collected together with approximately 0.5 mL of the surrounding separation medium and transferred into a new centrifuge tube. Cells were washed with cell washing solution and centrifuged at 400 × g for 10 min. When residual erythrocyte contamination was observed, red blood cells were removed using red blood cell lysis buffer according to the manufacturer’s protocol. Following erythrocyte lysis, cells were centrifuged at 250 × g for 5 min, washed again with cell washing solution, and centrifuged at 250 × g for 10 min.

Purified neutrophils were resuspended in pre-warmed complete IMDM medium and allowed to equilibrate for 30 min before subsequent experiments.

## Mouse Neutrophil Isolation

Mouse bone marrow neutrophils were isolated from femurs and tibias using the Mouse Bone Marrow Neutrophil Isolation Kit (P8550, Solarbio, China) according to the manufacturer’s instructions. Briefly, bone marrow cells were collected to prepare single-cell suspensions. The cell suspension was carefully layered onto the proprietary density-gradient separation medium supplied with the kit. The density-gradient system was prepared by sequentially adding 4 mL of reagent A and 2 mL of reagent C into a sterile 15 mL centrifuge tube, followed by gentle addition of the cell suspension onto the upper surface of the gradient interface. Samples were centrifuged at 500–1000 × g for 20–30 minutes at room temperature using a horizontal rotor.

After centrifugation, the granulocyte-containing layer was collected and transferred into a new centrifuge tube. Cells were washed with 10 mL cell washing solution by centrifugation at 400 × g for 10 minutes. When necessary, residual erythrocytes were removed using red blood cell lysis buffer for 5 minutes, followed by centrifugation at 250 × g for 5 minutes. After washing, cells were resuspended in 10 mL cell washing solution and centrifuged at 250 × g for 10 minutes. The final neutrophil-containing cell suspension was resuspended for subsequent experiments.

Mouse peripheral blood neutrophils were isolated using the Mouse Peripheral Blood Neutrophil Separation Kit (P9201, Solarbio, China) according to the manufacturer’s protocol.

# NET-associated Extracellular DNA Preparation

Human neutrophils were seeded into 24-well plates at a density of 1.8 × 10⁶ cells/well. Cells were stimulated with 50 nM phorbol 12-myristate 13-acetate (PMA; Sigma-Aldrich) for 4 hours to induce extracellular DNA release. Following stimulation, culture media were removed and wells were gently washed with RPMI 1640. Extracellular DNA-associated structures were collected by washing with cold PBS. Samples were centrifuged at 1000 × g for 10 minutes at 4°C, followed by centrifugation at 20 × g for 5 minutes. The resulting supernatants containing extracellular DNA-associated complexes were collected for subsequent experiments or stored at -80°C.

# Quantification of Cell-Free DNA

Cell-free DNA (cfDNA) levels in plasma and culture supernatants were measured using the Quant-iT PicoGreen dsDNA Assay Kit (Thermo Fisher Scientific).

Briefly, 50 μL of diluted samples or DNA standards (0–1000 ng/mL) were added to 96-well plates. Equal volumes of PicoGreen reagent were added, and samples were incubated in the dark according to the manufacturer's instructions. Fluorescence intensity was measured using excitation and emission wavelengths of 480 nm and 520 nm, respectively.

# Detection of MPO-DNA and NE-DNA Complexes

NET-associated MPO-DNA and NE-DNA complexes were quantified using commercial capture ELISA kits (JianglaiBio). Plasma samples and cell culture supernatants were analyzed according to the manufacturer's protocols. MPO-DNA and NE-DNA complexes were interpreted as NET-associated extracellular DNA markers reflecting neutrophil-derived extracellular DNA release. Measurements were performed in duplicate.

# Immunofluorescence Staining

Neutrophils were cultured on poly-L-lysine-coated glass coverslips. Cells were fixed with 4% paraformaldehyde for 30 minutes at room temperature and permeabilized according to experimental requirements. After blocking, samples were incubated overnight with primary antibodies at 4°C, followed by incubation with fluorescent secondary antibodies. Coverslips were mounted using DAPI-containing fluorescence mounting medium. NET-associated fluorescence signals were evaluated by extracellular DNA co-localization with myeloperoxidase (MPO), neutrophil elastase (NE), and citrullinated histone H3 (Cit-H3). Images were acquired using a Nikon C2 confocal microscope. Fluorescence intensity was quantified using ImageJ software.

# Multiplex Immunofluorescence

Multiplex immunofluorescence staining was performed using a three-color tyramide signal amplification (TSA)-based fluorescence kit (Hunan Aifang Biotechnology Co., Ltd.) according to the manufacturer's instructions. After sequential staining, fluorescence images were acquired using confocal microscopy. Image processing and quantitative analysis were performed using NIS Elements BR 5.01 software and ImageJ.

# Histological Analysis

Mouse tissues were fixed in 10% neutral-buffered formalin, embedded in paraffin, and sectioned at 5 μm thickness. Sections were stained with hematoxylin and eosin (H&E) for pathological evaluation or toluidine blue for mast cell visualization. Six randomly selected high-power fields (40× magnification) were examined for each sample. Mast cell numbers were quantified by an investigator blinded to experimental groups.

# Primary Neutrophil Stimulation Experiments

Mouse bone marrow neutrophils were cultured with DNase I, TFPI, sodium heparin, dexamethasone, or PBS control. For IgE-mediated stimulation experiments, neutrophils were sensitized with 0.1 μg/mL DNP-specific IgE for 16 hours. Cells were subsequently stimulated with 1 μg/mL DNP-HSA for 4 hours. For NET-associated extracellular DNA preparation, PMA stimulation was used as a positive control where indicated.

# ELISA Analysis

Commercial ELISA kits (JianglaiBio, China) were used to quantify plasma levels of MPO-DNA complexes, NE-DNA complexes, tissue factor (TF), tissue factor pathway inhibitor (TFPI), D-dimer, fibrin degradation products (FDP), histamine, interleukin-4 (IL-4), interleukin-10 (IL-10), and TNF-α according to the manufacturer's instructions. Absorbance was measured using a microplate reader (Rayto RT-6100). TF concentrations were interpreted as TF protein expression levels and not as direct measurements of functional TF activity.

# Quantitative Real-Time PCR

Total RNA was extracted from cultured neutrophils using TRIzol reagent (Invitrogen). RNA concentration and quality were assessed before reverse transcription. Complementary DNA was synthesized using the ImProm-II Reverse Transcription System (Promega). Quantitative real-time PCR was performed using SYBR Green PCR Master Mix (Applied Biosystems) on an ABI 7300 PCR system. Relative gene expression levels were normalized to β-actin. Expression changes were calculated using the 2^−ΔΔCt method. Primer sequences are listed in Supplementary Table 3.

# Additional Statistical Details

All experiments were performed using independent biological replicates unless otherwise indicated. Data distribution was evaluated using the Shapiro–Wilk test.

For normally distributed data, results are expressed as mean ± standard deviation (SD). For non-normally distributed data, results are expressed as median with interquartile range (IQR). Two-group comparisons were performed using Student’s t test or Mann–Whitney U test. Multiple-group comparisons were performed using one-way ANOVA with Tukey’s correction or Kruskal–Wallis analysis with Dunn’s correction.

Repeated temperature measurements in PSA models were analyzed using two-way repeated-measures ANOVA with Šidák correction. Correlation analyses were performed using Pearson or Spearman methods according to data distribution. A two-sided P <0.05 was considered statistically significant. For transcriptomic analyses, adjusted P values after multiple testing correction were used.

**Supplementary Table 1. Clinical characteristics of 323 patients with anaphylaxis.**

| **Clinical characteristics** | Mild (n=241) | Moderate (n=56) | Severe (n=26) |
| --- | --- | --- | --- |
| Age (year), mean ± SD | 38.34±15.542 | 37.70±16.483 | 38.12±18.378 |
| Sex, n (%) |  |  |  |
| Female | 145 (60.17) | 37 (66.07) | 15 (57.69) |
| Male | 96(39.83) | 19 (33.93) | 11 (42.31) |
| Allergen category |  |  |  |
| drug | 118 (48.96) | 27 (48.21) | 10 (38.46) |
| food | 65 (26.97) | 16(28.57) | 9 (34.62) |
| insect stings | 13 (5.39) | 1 (1.79) | 1 (3.85) |
| Others like exercise /alcohol | 10 (4.15) | 1 (1.79) | 2 (7.69) |
| Unknown | 35 (14.52) | 17 (19.64) | 7 (15.38) |
| White blood cell(*10^9/L) | 7.22 (5.92,8.60) | 10.31 (9.33,11.48) | 10.20 (8.95,12.51) |
| Neutrophil(*10^9/L) | 5.42 (4.19,6.36) | 8.61 (7.40,9.07) | 7.66 (6.94,9.86) |
| Lymphocyte(*10^9/L) | 1.52±0.74 | 1.59±0.65 | 1.48±0.76 |
| Monocyte(*10^9/L) | 0.42±0.23 | 0.53±0.21 | 0.43±0.29 |
| Eosinophils(*10^9/L) | 0.11±0.22 | 0.16± 0.33 | 0.13±0.35 |
| Platelet(*10^9/L) | 238.00 (188.75,292.25) | 250.00 (206.00,284.00) | 254.00 (229.00,340.50) |
| Hemoglobin(g/L) | 128.60±13.10 | 130.52±15.75 | 118.65±18.76 |
| D-dimer(mg/L) | 1.84±1.81 | 6.82±1.44 | 11.71±3.25 |
| FDP(ng/ml) | **19.72±1.79** | **28.71±2.63** | **34.68±2.94** |

Abbreviations: FDP, fibrin degradation products; SD, Standard Deviation.

**Supplementary Table 2. Information of antibodies.**

| Antibodies | Source | Catalog Number | Dilutions |
| --- | --- | --- | --- |
| Immunofluorescence | | | |
| cit-H3 | abcam | ab246514 | 1:200 |
| myeloperoxidase | abcam | ab134132 | 1:200 |
| TF | Sekisui Diagnostics | no. 4509 | 1:200 |
| neutrophil elastase | abcam | ab254178 | 1:100 |

**Supplementary Table 3. Sequence of Primers and real-time RT-PCR conditions.**

| Gene name | Forward primer (5’-3’) | Reverse primer (5’-3’) |
| --- | --- | --- |
| Mice β-actin | GCAGGAGTACGATGAGTCCG | ACGCAGCTCAGTAACAGTCC |
| Mice IL-4 | TACCAGGAGCCATATCCACGGATG | TGTGGTGTTCTTCGTTGCTGTGAG |
| Mice IL-6 | AGACTTCCATCCAGTTGCCTTCTTG | CATGTGTAATTAAGCCTCCGACTTGTG |
| Mice IL-10 | CTATGCTGCCTGCTCTTACTGACTG | GAGTCGGTTAGCAGTATGTTGTCCAG |
| Mice IL-13 | CAACGGCAGCATGGTAT | GCAATATCCTCTGGGTCCT |
| Mice TNF-α | GAAACACAAGATGCTGGGA | TTGCAGAACTCAGGAATGG |
| Mice TF | CAATGAATTCTCGATTGATGTGG | GGAGGATGATAAAGATGGTGGC |
| Mice TFPI | ACTGTGTGTCTGTTGCTTAGCC | GTTCTCGTTCCCTTCACATCCC |
| Human DUSP2 | TGTCCCGATCTGTGCTCTGAGG | CACTGATCTCCACCATCTGGTTGTC |
| Human DDIT4 | CTCGTCGTCGTCCACCTCCTC | GGCTTACCAACTGGCTAGGCATC |
| Human ALOX15 | CGGTATGTGGAAGGAATCGTGAGTC | ATCTGGAGAGAAGCCTGGTGGAAG |
| Human GZMB | GTGCGGTGGCTTCCTGATACG | CCTGCACTGTCATCTTCACCTCTTG |
| Human ITGB3 | CGGAAGGCTGGCAGGCATTG | TGTTGAGGCAGGTGGCATTGAAG |
| Human IL18R1 | GTGCCTGGAGGAGCTGTTGTTG | GTTCGTCTCTACCTGGCTTGACTG |
| Human CCL4 | GAAGCTCTGCGTGACTGTCCTG | ATTCACTGGGATCAGCACAGACTTG |
| Human MPL | CCAAGTCAGCAGCCAAGATGTCTC | ACGCACTTCCTCCTGGTCTGG |
| Human BCL2A1 | GTCCGTAGACACTGCCAGAACAC | TAGGTGTGTGATTGTGCCATTTCCC |
| Human LRP1 | AGTATCGGAGTGGCAGTGTCTACC | TGTTGTCCAGGCAATCGTTGTCTC |
